# An investigation of crosstalk between Wnt/β-catenin and transforming growth factor-β signaling in androgenetic alopecia

**DOI:** 10.1097/MD.0000000000004297

**Published:** 2016-07-29

**Authors:** Gui-Qing Lu, Zhi-Bo Wu, Xiao-Yan Chu, Zhi-Gang Bi, Wei-Xin Fan

**Affiliations:** a Department of Dermatology, The First Affiliated Hospital of Nanjing Medical University; b Department of Dermatology, BenQ Medical Center, Nanjing Medical University; c Department of Dermatology, Friendship Plastic Surgery Hospital, Nanjing Medical University, Nanjing, P.R. China.

**Keywords:** androgenetic alopecia, dermal papilla cell, hair follicle, SB431542, TGF-β, Wnt, XAV939

## Abstract

**Background:**

Wnt and transforming growth factor-β (TGF-β) signaling pathways are known to be involved in the pathogenesis of androgenetic alopecia (AGA). However, the way that Wnt and TGF-β signaling is altered in patients with AGA and whether there exists a crosstalk between them in pathogenetic process of AGA remain unclear.

**Objectives:**

To investigate the expression of Wnt and TGF-β signaling and the crosstalk between these 2 signaling pathways in AGA.

**Methods:**

Fifteen male patients with AGA were recruited for our research. Fifteen scalp specimens of the balding were collected from frontal areas, and 9 nonbalding were collected from occipital areas. We analyzed the expression and activation of downstream Wnt and TGF-β signaling molecules in both balding and nonbalding hair follicles isolated from scalp specimens. Furthermore, we evaluated the activation of Wnt and TGF-β signaling after either of them was blocked with the inhibitor in balding and nonbalding dermal papilla (DP) cells.

**Results:**

Compared with the nonbalding counterparts, the mRNA level of *Wnt10a* and *LEF1* was decreased. But *TβRI* and *TβRII*, and the protein expression of TGF-β1 was elevated in balding hair follicles. To investigate the crosstalk between Wnt and TGF-β signaling, we used SB431542 to inhibit the TGF-β signaling in balding DP cells and found that SB431542 significantly attenuated the phosphorylation of Smad2 and Akt. However, the mRNA level of *Wnt10a*, *LEF1*, and the nuclear translocation of β-catenin was increased. On the other hand, we suppressed the Wnt signaling by XAV939 in nonbalding DP cells, which displayed that the level of β-catenin and *LEF1* was significantly inhibited; however, the level of active TGF-β1 and the phosphorylation of Smad2 and Akt were up-regulated.

**Conclusions:**

These data indicate that crosstalk between Wnt/β-catenin and TGF-β signaling pathways may exist as one of the important mechanisms contributing to AGA.

## 1 Introduction

Androgenetic alopecia (AGA) is a genetically determined, androgen induced, and age depended common hair loss disorder, and affects both men and women. Despite the fact that specific molecules regulated by androgen in the balding scalp are not fully clear, several cytokines, hormones, growth factors, enzymes, and neuropeptides are contributors to hair cycle control and may be involved in the pathogenesis of AGA.^[1–3]^ Wnt and transforming growth factor-β (TGF-β) ligands function in numerous developmental processes, and the alterations of both signaling pathways are associated with common pathologic conditions, including AGA.

The cell fate choice of stem cell of complex organs and tissues is regulated by microenvironment during the embryonic stage.^[4,5]^ Recent studies have revealed that Wnt/β-catenin signaling was required to trigger epidermal stem cell in the process of hair follicle formation, and it induced the hair follicle dermal papilla (DP) formation.^[6]^ The results from transgenic mice showed that stabilization of β-catenin could promote the process of placode maturity and development,^[7]^ while the genetic or pharmacologic ablation of β-catenin could cause the failure of hair follicles formation.^[6]^ Furthermore, crosstalk between androgen and Wnt/β-catenin signaling is thought to play an important role in AGA pathogenesis.^[8]^ It is also well demonstrated, in the DP cells and keratinocytes co-culture system, that the secretion of TGF-β1 induced by androgen is essential in DP cells from balding frontal scalp via influencing the epithelial cell growth.^[9]^ In addition, TGF-β1 modulates androgen receptor transactivation and androgen sensitivity in DP cells,^[10]^ which contributes to the pathology of AGA.

Several studies have shown that cooperation between TGF-β and Wnt signaling pathways plays an important role in controlling certain developmental events. Crosstalk between the TGF-β and Wnt signaling pathways participates in the formation of tooth eruption passage,^[11]^ the development of prostate cancer,^[12]^ wound healing,^[13]^ and tissue fibrosis in skeletal muscle and kidney.^[14]^ However, whether there is a crosstalk between these 2 important signaling pathways during AGA pathological procession is still unknown.

This study, therefore, explored the differences in the levels of Wnt (*Wnt10a*, *LEF1*, β-catenin) and TGF-β (TGF-β1, Smad2, Akt, *TβRI*, *TβRII*) signaling in balding versus nonbalding hair follicles isolated from 15 scalp specimens and DP cells. Furthermore, the study examined the activation of the Wnt/β-catenin signaling after the TGF-β signaling pathway was inhibited in balding DP cells, and also evaluated the activation of the TGF-β signaling after the Wnt/β-catenin signaling pathway was blocked in nonbalding DP cells. These data confirm that both regulations of Wnt/β-catenin and TGF-β signaling pathways are involved in pathologic process of AGA and support the hypothesis that crosstalk between Wnt/β-catenin and TGF-β signaling pathway may exist in the pathogenesis of AGA.

## 2 Materials and methods

### 2.1 Patient population and samples

The clinical data of patients who consented to this study in BenQ Medical Center were retrospectively analyzed. The study was performed after the minimal-risk institutional review board approval. Patients meeting the following criteria were included in the study: positive diagnosis as AGA (Hamilton–Norwood classifications III–V anterior), and exclusion of traumatic and inflammatory treatment. A total of 15 men, aging between 22 and 47 years (average 30 years), were involved in this study. Scalp specimens of the balding (frontal) areas were collected from all 15 patients with AGA, and specimens of the nonbalding (occipital) were collected from 9 of them.

### 2.2 Isolation and cultivation of DP cells

The isolation and cultivation of DP cells from balding and nonbalding scalps were carried out as previously described.^[15]^ In brief, we obtained skin specimens during plastic surgery and thoroughly washed them with D-Hanks buffer (containing 5% [v/v] penicillin and streptomycin). Then, we carefully removed the subcutaneous fat and connective tissue surrounding the collagen capsules by microscissors and digested the separated tissues with 0.1% collagenase type I (Sigma, St Louis, MO) at 37°C for 1 h. Afterward, the papillae were dissociated and cultured in DMEM/F-12 medium (Gibco, Paisley, UK) with 10 ng/mL basic fibroblast growth factor and 10% fetal bovine serum. Finally, we used 0.25% trypsin (Invitrogen, Carlsbad, CA) with 0.02% EDTA to digest DP cells and planted them into a new culture dish at 1 × 10^4^ cells/mL and maintained at 37°C under a humidified atmosphere of 95% O_2_ and 5% CO_2_ for 24 h prior to complement with serum-free DMEM/F-12 medium. The DP cells used in this research were in the second and third passages of subculture.

### 2.3 RT-qPCR

Human hair follicles were isolated from the balding and nonbalding scalps according to the method of Philpott et al.^[16]^ Total RNA of hair follicles and DP cells was isolated by using TRIzol Reagent (Invitrogen, Grand Island, NY). RNA was reverse-transcribed using the PrimeScriptTMRT reagent Kit (TaKaRa, Dalian, China). Real time quantitative PCR (RT-qPCR) was performed with the corresponding primers (Table 1) and the SYBR Premix Ex Taq II (Takara) by using StepOnePlus System (Applied Biosystems, Grand Island, NY). Gene expression was normalized by Glyceraldehyde-3-phosphate dehydrogenase and these data were analyzed using StepOne Software.

**Table 1:**
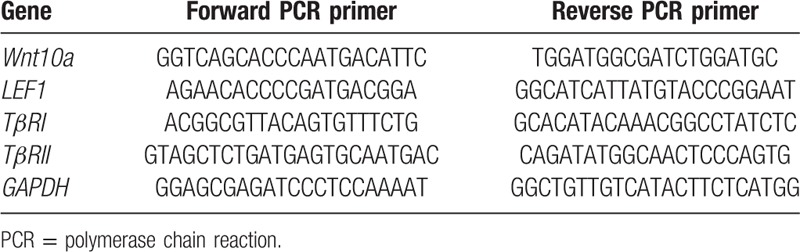
Primers designed for RT-qPCR.

### 2.4 ELISA

After collecting the medium of DP cells culture, we used ELISA assay kit (Quantikine human TGF-β1 immunoassay, R&D Systems, Minneapolis, MN) to measure the concentration of total (latent and active) and active TGF-β1, according to the manufacturer's instructions.

### 2.5 Immunofluorescence

DP cells (the second passages of subculture) grown on glass coverslips were fixed in 4% paraformaldehyde, incubated with phosphate-buffered saline (PBS) containing 100 mmol/L glycine, and washed with PBS. Cells were permeabilized in PBS containing 0.1% Triton X-100, blocked with 5% albumin for 1 h, and incubated with mouse monoclonal antibody against β-catenin (1:1000 dilution, Milipore, Billerica, MA) overnight. After several rounds of washing, goat anti-mouse-fluorescein or rabbit anti-mouse-fluorescein (1:2000 dilution, Thermo Fisher Scientific, Waltham, Massachusetts, USA) was added for 1 h, and the coverslips were washed and mounted using fluorescence microscopy.

### 2.6 Western blotting assay

Protein samples from hair follicles or cultured DP cells were extracted using RIPA buffer (Beyotime, Nanjing, JS, China). Equal amounts of protein were loaded in each lane, resolved on 10% sodium dodecyl sulfate poly-acrylamide gradient gels and transferred onto a 0.45-μm NC membrane (Milipore). The membrane was blocked with 5% bovine serum albumin and incubated with anti-p-Smd2, anti-Smd2, anti-p-Akt, anti-Akt, anti-β-catenin (1:1000; Milipore) or Laminin B1 (polyclonal antibody, 1:1000; Sigma) overnight at 4°C. Followed, then by anti-rabbit or anti-mouse IgG peroxidase conjugate. Immunoreactive bands were visualized by the Immobilon Western hemiluminescent HRP Substrate (Milipore).

### 2.7 Statistical analysis

A statistical analysis was performed using SPSS software version 17.0. These data were presented as the mean ± standard error of the mean from at least 3 independent experiments. Multiple group comparisons were made using 1-way analysis of variance. Differences between groups were considered statistically significant at *P* < 0.05.

## 3 Results

### 3.1 Wnt signaling is down-regulated in AGA

**Figure 1:**
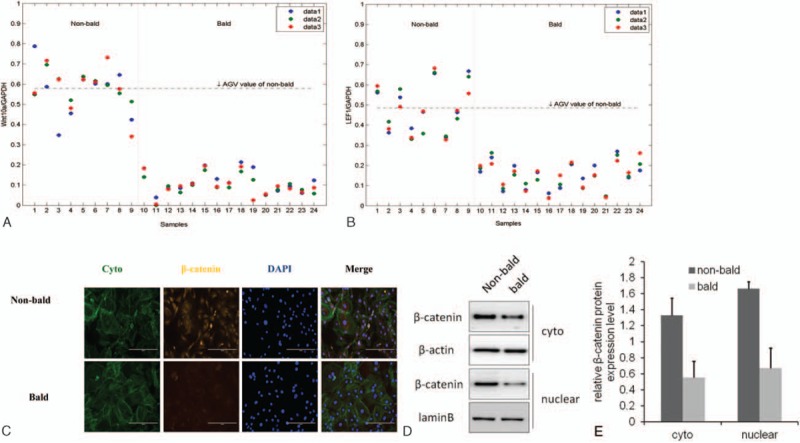
Activation of Wnt/β-catenin signaling in AGA pathology. Fifteen specimens of the balding hair follicles were collected from the frontal scalp (lanes 10–24), and 9 specimens of nonbalding hair follicles were collected from occipital scalp (lanes 1–9). The mRNA level of *Wnt10a* and *LEF1* was evaluated by RT-qPCR. The data showed that the average value of mRNA level of *Wnt10a* (A) and *LEF1* (B) in balding hair follicles was much lower than the nonbalding. DP cells were isolated and cultured from balding and nonbalding scalp, respectively, and the β-catenin expression in DP cells was assessed by immunofluorescence (C) and western blotting (D and E). The result turned out that the activity of β-catenin in balding DP cells was lower compared with the nonbalding, not only in the cytoplasm but also in the nuclear (C–E). The cellular localization of β-catenin (green) with immunofluorescence was shown by using cyto tracer to stain cytoplasm and DAPI (4’, 6-diamidino-2-phenylindole) to stain nuclei, scar bar = 200 μm (n = 3 per group, data are presented as mean ± standard error of the mean). AGA = androgenitic alopecia, DAPI = 4′,6-diamidino-2-phenylindole, DP = dermal papilla.

To test whether the Wnt signaling is altered during AGA pathology, we performed the RT-qPCR analysis to evaluate the mRNA level of *Wnt10a* and *LEF1* (Fig. 1A and B) in hair follicles. The data showed that the average value of mRNA level of *Wnt10a* and *LEF1* in balding hair follicles from the frontal scalp was much lower than that in nonbalding from the occipital scalp. Furthermore, we isolated and cultured DP cells from balding and nonbalding scalp, respectively. We assessed the β-catenin expression by immunofluorescence (Fig. 1C) and by western blotting (Fig. 1D and E), and found out that the activity of β-catenin in balding DP cells was lower compared with the nonbalding DP cells, not only in the cytoplasm but also in the nuclear.

### 3.2 TGF-β signaling is up-regulated in AGA

**Figure 2:**
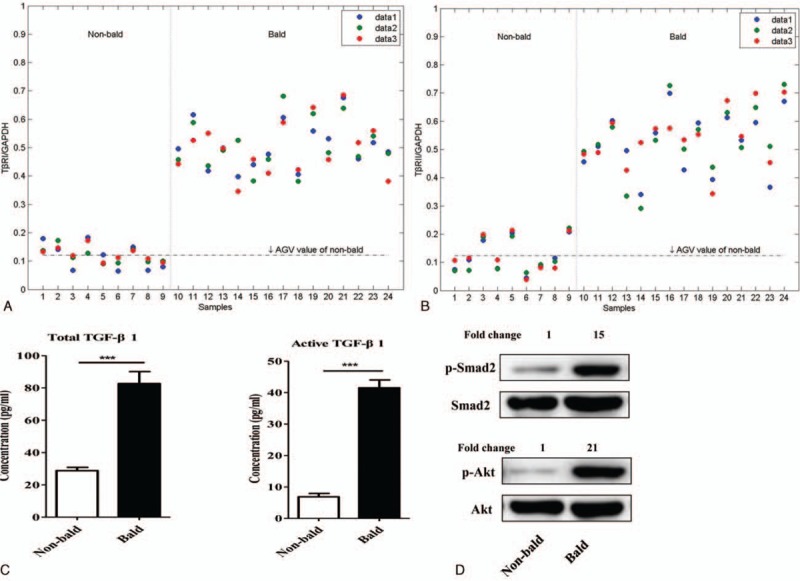
Activation of TGF-β signaling in AGA pathology. To confirm the alteration of TGF-β signaling in AGA, the mRNA level of *TβRI* and *TβRII*, protein concentration of total and active TGF-β1, and protein level of p-Smad2, Smad2, p-Akt, and Akt were measured by RT-qPCR, ELISA, and western blotting methods, respectively. Compared with the nonbalding, the average mRNA level of *TβRI* (A) and *TβRII* (B) and the protein concentration of total and active TGF-β1 (C) were significantly increased in balding hair follicles. The phosphorylation of Smad2 and Akt was significantly up-regulated in the balding DP cells than the nonbalding (D) (n = 3 per group, data are presented as mean ± standard error of the mean, ∗∗∗*P* < 0.001). AGA = androgenitic alopecia, DP = dermal papilla, TGF-β = transforming growth factor-β.

To confirm the alteration of TGF-β signaling in AGA, we measured the mRNA level of *TβRI* and *TβRII* and the concentration of total and active TGF-β1 in hair follicles, and the level of key mediators in TGF-β signaling in cultured DP cells (Fig. 2A–D). In the hair follicles, the average mRNA level of *TβRI* and *TβRII* in balding hair follicles was much higher than that in the nonbalding (Fig. 2A and B). Consistent with the expression of TGF-β receptors, the concentration of total and active TGF-β1 were significantly increased in balding hair follicles compared with the nonbalding (Fig. 2C). To investigate the activation of TGF-β signaling pathway, we selected 2 important mediators in this pathway, Smad2 and Akt. The western blotting results showed that the phosphorylation of Smad2 and Akt was up-regulated in the balding DP cells (Fig. 2D).

### 3.3 Wnt signaling is activated in balding DP cells by treatment of SB431542

**Figure 3:**
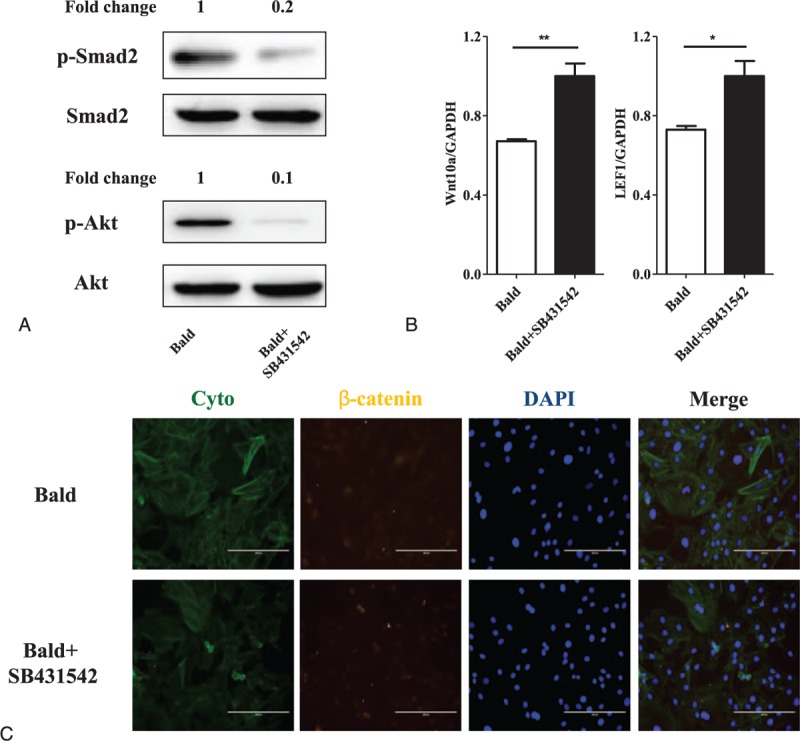
Activation of Wnt/β-catenin signaling in balding DP cells treated with SB431542. Balding DP cells were cocultured with SB431542 (at a concentration of 20 μM) for 4 d to inhibit the TGF-β signaling and the activation of Wnt signaling was subsequently evaluated by the methods of western blotting and RT-qPCR. The significant down-regulation of phosphorylation level of Smad2 and Akt confirmed that SB431542 was sufficient to inhibit the TGF-β signaling in balding DP cells (A). The RT-qPCR data showed that mRNA level of *Wnt10a* and *LEF1* was significantly increased in balding DP cells after SB431542 treatment (B). The β-catenin expression was further detected by immunofluorescence (C). The cellular localization of β-catenin (green) was shown by using cyto tracer to stain cytoplasm and DAPI (4’, 6-diamidino-2-phenylindole) to stain nuclei, scar bar = 200 μm. Results revealed that the total β-catenin expression was not significantly altered but the β-catenin nuclear accumulation was remarkably increased (n = 3 per group, data are presented as mean ± standard error of the mean, ^∗^*P* < 0.05, ^∗∗^*P* < 0.01 compared with the bald). DAPI = 4′,6-diamidino-2-phenylindole, DP = dermal papilla, TGF-β = transforming growth factor-β.

To investigate whether there is a crosstalk between Wnt/β-catenin and TGF-β signaling in AGA, we treated balding DP cells with SB431542 (at a concentration of 20 μM for 4 days) to inhibit the TGF-β signaling, and evaluated the activation of Wnt signaling subsequently by the methods of western blotting and RT-qPCR. The significant down-regulation of phosphorylation level of Smad2 and Akt confirmed that SB431542 was sufficient to inhibit the TGF-β signaling in balding DP cells (Fig. 3A). The RT-qPCR data showed that mRNA level of *Wnt10a* and *LEF1* was significantly increased in balding DP cells after the SB431542 treatment (Fig. 3B). We further detected the β-catenin expression by immunofluorescence, and the results revealed that the total β-catenin expression was not significantly altered, but the β-catenin nuclear accumulation was remarkably increased in the SB431542 treated-DP cells (Fig. 3C).

### 3.4 TGF-β signaling is activated in nonbalding DP cells by treatment of XAV939

**Figure 4:**
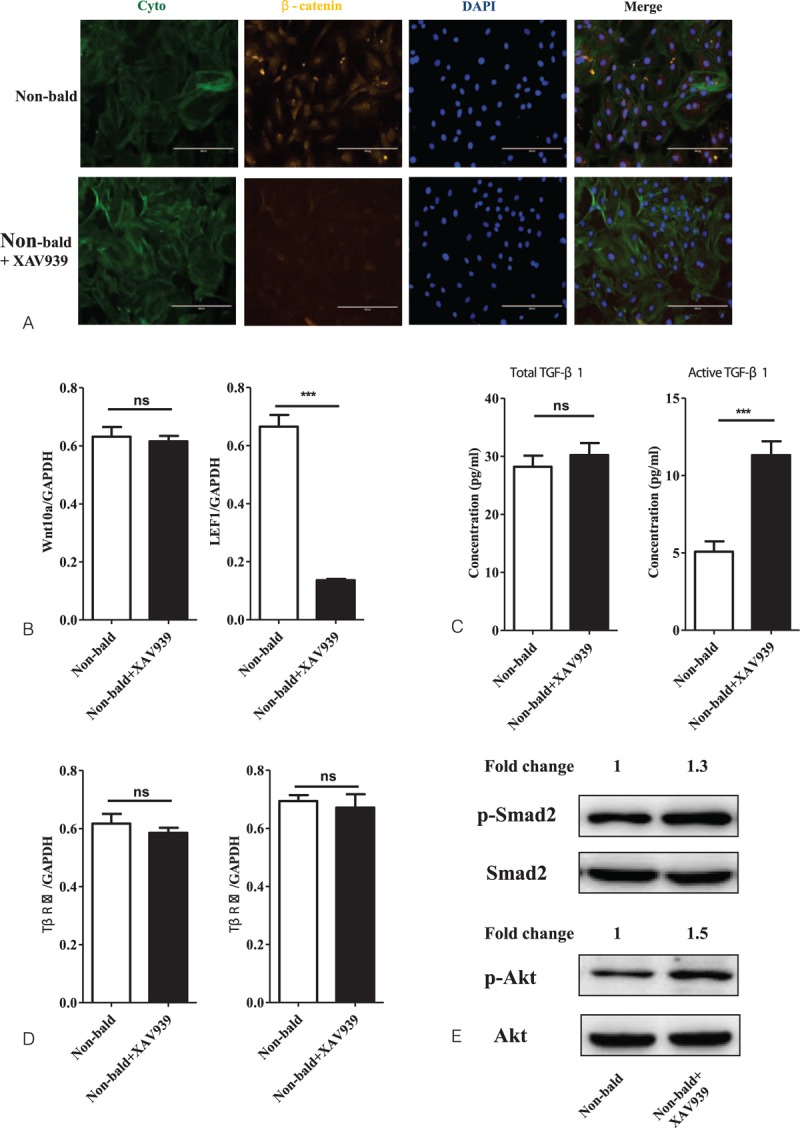
Activation of TGF-β signaling in nonbalding DP cells treated with XAV939. The nonbalding DP cells were treated with XAV939 (at a concentration of 20 μM) for 4 d to inhibit the Wnt/β-catenin signaling, and activation of TGF-β signaling was assessed afterward. The expression of β-catenin (A) and mRNA level of *LEF1* (B) was significantly inhibited by XAV939, but the mRNA level of *Wnt10a* (B) was not altered. The protein concentration of total and active TGF-β1, the mRNA level of *TβRI* and *TβRII*, and the protein expression of p-Smad2, Smad2, p-Akt, and Akt were tested by ELISA, RT-qPCR, and western blotting, respectively. With the significant suppression of Wnt signal-mediated transcription in nonbalding DP cells, XAV939 significantly increased the concentration of active TGF-β1 (C) but without affecting the total TGF-β1 (C) and the expression of *TβRI* and *TβRII* (D). The phosphorylation level of Smad2 and Akt was up-regulated with the increased phosphorylation of Smad2 and Akt (E) (n = 3 per group, data are presented as mean ± standard error of the mean and ^∗∗∗^*P* < 0.001 compared with the nonbald). DP = dermal papilla, ns = nonsignificant difference, TGF-β = transforming growth factor-β.

To determine more about the interaction between Wnt/β-catenin and TGF-β signaling in AGA, we further isolated and cultured DP cells from tissues and treated nonbalding DP cells with XAV939 (at a concentration of 20 μM for 4 days) to inhibit the Wnt/β-catenin signaling, and assessed activation of TGF-β signaling afterward. As the data showed, the expression of β-catenin and the mRNA level of *LEF1* were significantly inhibited in nonbalding DP cells by supplement with XAV939 (Fig. 4A and B), but the mRNA level of *Wnt10a* was not altered (Fig. 4B). To evaluate the activation of TGF-β signaling, we tested the protein concentration of total and active TGF-β1 and the mRNA level of *TβRI* and *TβRII*. It turned out that XAV939 significantly increased the concentration of active TGF-β1 (Fig. 4C), but without affecting the total TGF-β1 (Fig. 4C) and the expression of *TβRI* and *TβRII* (Fig. 4D). In addition, we detected the phosphorylation level of Smad2 and Akt with a result of up-regulation of phosphorylation of Smad2 and Akt (Fig. 4E).

## 4 Discussion and conclusions

The regulation activity of androgen on hair growth and cycling varies on different body sites in puberty males, which promotes beard growth but inhibits hair growth in frontal and vertical scalp, known as “androgen paradox.” More and more evidence proves that androgen induced hair growth suppression is tightly related to Wnt/β-catenin signaling, which promotes perspective crosstalk occurring between androgen and Wnt/β-catenin signaling in AGA pathogenesis.^[8,17]^ For instance, dihydrotestosterone (DHT)-treated DP cells from AGA inhibits the Lymphoid enhancer factor/T-cell factor induced transcriptional activity^[18]^ and hair follicle stem cell differentiation,^[8,19]^ while Wnt signaling reactivation will restore the DHT induced hair follicle stem cell differentiation.^[8]^

In this study, we confirmed that the activation of Wnt/β-catenin signaling was indeed down-regulated in the AGA pathogenetic process (Fig. 1). On the other hand, TGF-β signaling is also an important androgen-inducible pathway in AGA development, not only androgen regulates TGF-β transcription^[20]^ but also TGF-β signaling promotes the AGA development^[10,21]^ and TGF-β1 deficiency delays catagen progression^[22]^ and perifollicular fibrosis in AGA.^[23]^ Furthermore, we also demonstrated that the TGF-β signaling was remarkably up-regulated during the AGA pathogenesis (Fig. 2). In the present study, the result of the up-regulation of TGF-β signaling with simultaneity of down-regulation of Wnt/β-catenin in AGA, which triggers the hypothesis that there might exit a crosstalk between the Wnt/β-catenin and TGF-β signaling pathways during the development of AGA. To demonstrate the influence of TGF-β signaling on Wnt/β-catenin signaling, we isolated and cultured DP cells from frontal scalp of AGA patients, then treated them with SB431542 (TGF-β/Smad signaling inhibitor) to depress the TGF-β signaling. Then we investigated the activation of Wnt/β-catenin signaling (Fig. 3). The results revealed that the TGF-β signaling was significantly inhibited in balding DP cells treated with SB431542 (at a concentration of 20 μM) for 4 days, meanwhile the activation of Wnt/β-catenin signaling was significantly increased, not only the up-regulation of the expression of *Wnt10a* and *LEF1* but also the β-catenin nuclear accumulation. It suggests that the TGF-β signaling be involved in the Wnt/β-catenin regulation during AGA development. For further research, we used XAV9399 (Wnt/β-catenin signaling inhibitor) to block the Wnt/β-catenin signaling in cultured nonbalding DP cells, which were isolated from occipital scalp, then evaluated the activation of TGF-β signaling (Fig. 4). The data showed that XAV939, which treated nonbalding DP cells (at a concentration of 20 μM for 4 days), could strongly inhibit the Wnt/β-catenin signaling by reducing the β-catenin nuclear accumulation and *LEF1* expression, while the TGF-β signaling was up-regulated with the increased concentration of active TGF-β1 and phosphorylation of Smad2 and Akt. It indicated that the Wnt/β-catenin might also contribute to TGF-β signaling regulation during AGA development.

Wnt/β-catenin and TGF-β/Smad signaling pathways are involved in the pathogenesis of AGA. Our clinical data are consistent with this view. In addition, the results of the inhibition of Wnt/β-catenin signaling resulting in up-regulation of TGF-β signaling and inhibition of TGF-β signaling resulting in up-regulation of Wnt/β-catenin indicate that there may exist a crosstalk between these 2 important signaling pathways, which would add more knowledge to understand the whole picture of mechanism that regulates AGA development. However, which cross points are involved between these 2 signaling pathways and how they interact with each other, needs to be explored more thoroughly in the future.

The available treatment methods of AGA mainly include drug therapy and hair transplantation. Due to some unacceptable side effects of the oral drugs and the limited hair follicle units of donor, it is difficult to carry out these therapies extensively and effectively. An understanding of the Wnt/β-catenin and TGF-β/Smad signaling pathways and the crosstalk between them involved in AGA may facilitate discovery of pharmacophores that modulate these pathways. Our data might have therapeutic implications for the development of anabolic drugs, for example, the combination of a Smad2 inhibitor, a TGF-β1 antagonist, or *TβRII* inhibitor might be effective to prevent male pattern baldness.

## Acknowledgments

We want to thank our many colleagues who have helped to achieve the clinical specimens and those lab researchers who have helped make the experiment complete.
